# Prognostic factors analysis of diffuse midline glioma

**DOI:** 10.1007/s11060-024-04605-6

**Published:** 2024-02-21

**Authors:** Jing Jiang, Wen-bin Li, Shao-wen Xiao

**Affiliations:** 1 Department of Neuro-oncology, Cancer Center, Beijing Tiantan Hospital, Capital Medical University, Beijing, 100071 China; 2 Department of Radiation Oncology, Beijing Tsinghua Changgung Hospital, School of Clinical Medicine, Tsinghua University, Beijing, 102218 China; 3 Department of Radiation Oncology, Peking University Cancer Hospital & Institute, 52 Fucheng Road, Haidian District, Beijing, 100142 China

**Keywords:** Glioma/ diffuse midline glioma, Radiotherapy, Temozolomide, Prognosis

## Abstract

**Purpose:**

This study retrospectively analyzes cases of diffuse midline glioma treated with radiotherapy, with the aim of investigating the prognosis of the tumor and its influencing factors.

**Methods:**

From January 2018 to November 2022, we treated 64 patients who were pathologically diagnosed with diffuse midline glioma. Among them, 41 underwent surgical resection, and 23 underwent biopsy procedures. All patients received postoperative radiotherapy. We followed up with the patients to determine the overall survival rate and conducted univariate and multivariate analyses on relevant indicators.

**Results:**

The median survival time for the entire patient group was 33.3 months, with overall survival rates of 92.9%, 75.4%, and 45.0% at 1 year, 2 years, and 3 years, respectively. Univariate and multivariate analyses indicated that older patients had a better prognosis.

**Conclusion:**

Patient age is an independent prognostic factor for patients with diffuse midline glioma undergoing radiation therapy.

## Background

The H3F3A gene mutation was first discovered in pediatric glioblastoma in 2012 [1]. Subsequent research found that H3K27M mutations are frequently observed in pediatric diffuse pontine gliomas and non-brainstem glioblastomas [2–3]. Gliomas with midline structures accompanied by H3K27M mutations were introduced as a new independent tumor subtype in the 4th edition of the WHO classification of central nervous system tumors in 2016, and were defined as WHO grade 4 DMG(Diffuse midline glioma) [4]. In 2018, cIMPACT-NOW [5] provided an interpretation of the definition of DMG with H3K27M mutations and proposed strict diagnostic criteria, stating that the tumor must exhibit diffuse growth (i.e., infiltrative), midline structures (thalamus, brainstem, spinal cord, etc.), histological morphology, and gliomas with H3K27M mutations. The 5th edition of the WHO classification of central nervous system (CNS) tumors in 2021 defined this malignant tumor as “diffuse midline glioma, H3K27 altered” (reflecting a variety of potential molecular and epigenetic changes) [6]. Patients with DMG accompanied by H3K27M alterations exhibit differences in disease progression, treatment, and prognosis. This study explores the age, histopathological grade, and site of onset characteristics and prognosis-related factors of patients with DMG accompanied by H3K27M alterations, aiming to provide more data references for the standardized and individualized precision treatment of such patients.

## Materials and methods

### Patients

Imaging studies indicated that the lesions were located in midline structures, including the thalamus, brainstem, cerebellum, and spinal cord. All patients in the cohort underwent radiotherapy. Histopathological examination revealed gliomas, and immunohistochemistry showed positive expression of H3K27M. The study included 64 patients, none of whom had severe cardiovascular or cerebrovascular diseases, nor any other malignancies. Hematological assessments showed hemoglobin levels ≥ 110 g/L, white blood cell (WBC) counts ≥ 4.0 × 10^9/L, platelet counts ≥ 100 × 10^9/L, alanine aminotransferase (ALT) ≤ 40 U/L, aspartate aminotransferase (AST) ≤ 40 U/L, and levels of urea, creatinine, and total bilirubin were less than 1.5 times the upper limit of normal.

The study population consisted of 38 males and 26 females, aged between 6 and 56 years, with a median age of 32 years. The disease was located in the spinal cord in 19 cases and intracranially in 45 cases. Of these, 41 patients underwent surgery, 23 underwent biopsy, 35 received concurrent oral temozolomide chemotherapy during radiation therapy, and 29 underwent radiation therapy alone.

### Radiotherapy and chemotherapy

The patient’s position was fixed using a thermoplastic film or vacuum cushion, followed by CT scan simulation positioning. The radiotherapy planning system was used for digital transmission and three-dimensional reconstruction of images. The visible tumour lesions in the images were delineated, based on MRI T1 enhanced images, T2 weighted images, and FLAIR images to determine the range of the tumour lesion (Gross target volume, GTV). The potential clinical areas that might be invaded were considered as the treatment target area (Clinical target volume, CTV, the median total dose of radiotherapy was 54 Gy. In the concurrent chemotherapy group, temozolomide was orally administered at a dose of 75 mg/m^2^/day during radiotherapy. Regular monitoring of the patient’s blood routine and liver and kidney function was maintained until the end of radiotherapy.

### Statistical analysis

A database was established using SPSS 17.0 statistical software. Univariate analysis was performed using the Kaplan-Meier method, and significant differences were tested using the two-tailed Logrank test. A stepwise regression multivariate analysis was conducted using the Cox regression model to evaluate independent prognostic factors.

## Results

**Fig. 1:**
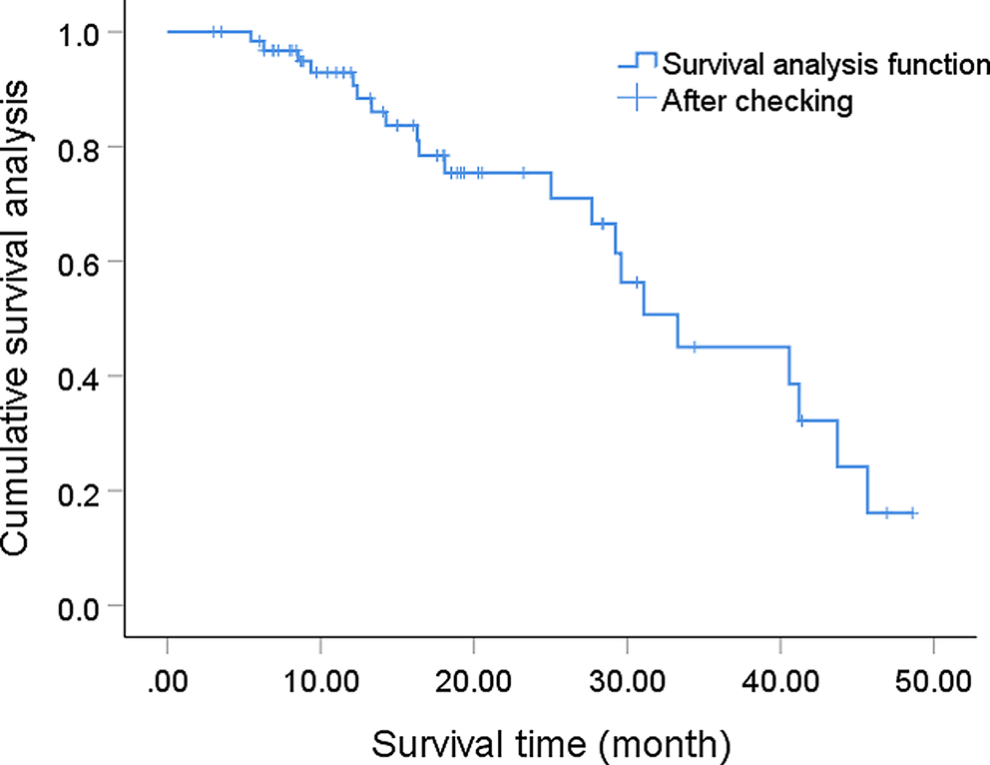
Survival curve for the entire patient group

The follow-up of this study was concluded in February 2023, with a median survival time of 33.3 months among the entire patient group. The overall survival rates for the first, second, and third years were 92.9%, 75.4%, and 45.0%, respectively, as shown in Fig. 1. Stratified analysis was performed on the entire patient group according to different factors. Numerical variables were grouped based on the median, such as age grouped by the median age of 32 years, Ki67 index grouped by the median of 27, and histopathology grouped by grade 4 and below.

### Univariate analysis

**Fig. 2:**
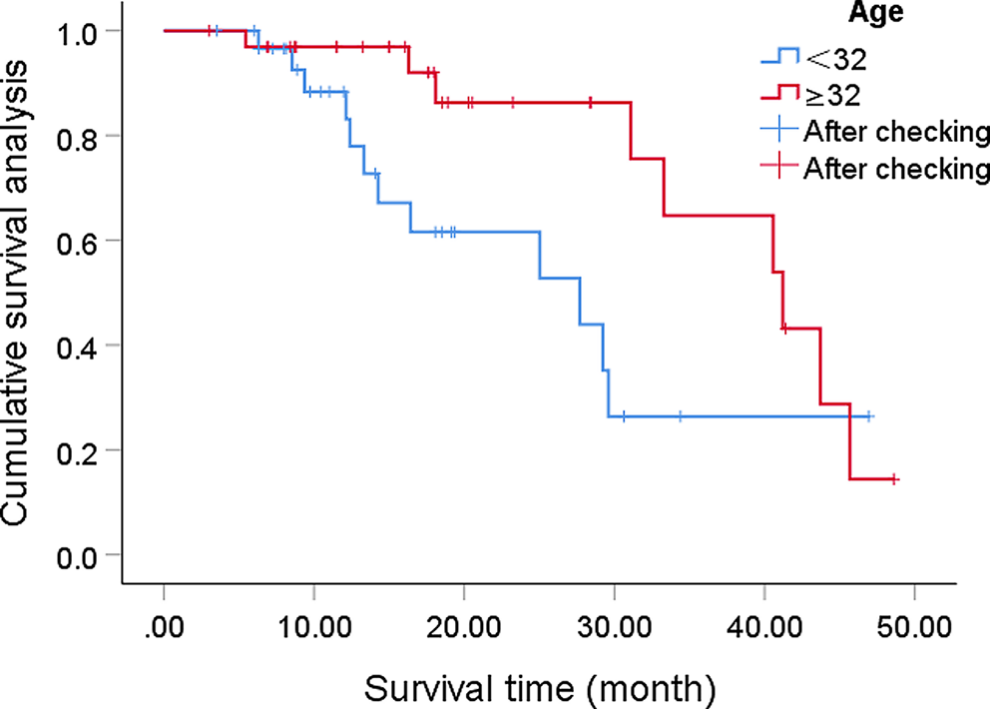
According to the stratified survival curve by age, patients under the age of 32 have a poorer prognosis, *P* = 0.033

**Fig. 3:**
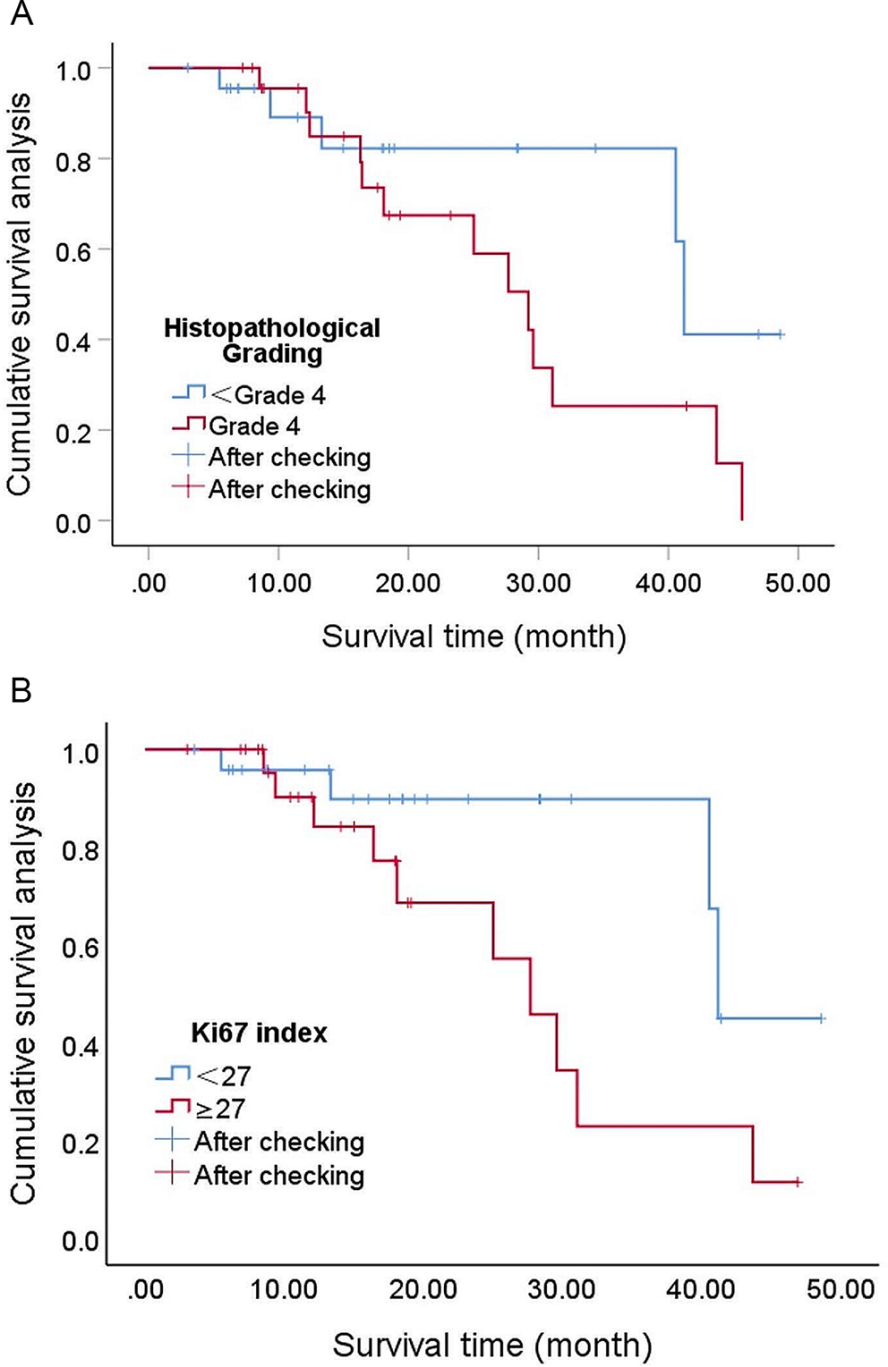
**A** Patients with a tissue grade of 4 have a poorer prognosis, *P* = 0.083. **B** The Ki67 index, stratified according to a median value of 27, indicates that a higher value corresponds to a worse prognosis, *P* = 0.060

Univariate analysis showed that the prognostic factor affecting the post-radiation treatment of DMG patients was the patient’s age, with younger patients having a worse prognosis, as shown in Fig. 2. The histopathological grading and the level of Ki67 index were statistically significant, as shown in Fig. 3. However, factors such as gender, surgical method, and whether concurrent temozolomide chemotherapy was used did not show statistically significant effects, as shown in Table 1.

**Table 1:** Univariate analysis of factors influencing the prognosis of diffuse midline glioma

Group Category	Numbers	Survival Rate(%)				χ^2^ value	P value
		1 year		2 year	3 year		
Gender						11.370	0.242
Male	38	91.1	69.6		35.4		
Female	26	96.0	84.0		58.8		
Age (Years)						4.565	**0.033**
< 32	31	88.3	61.5		26.4		
≥ 32	33	96.9	86.3		14.4		
Histopathological Grading						3.009	0.083
< Grade 4	23	89.1	82.2		82.2		
Grade 4	24	95.5	67.4		25.3		
Ki67 index						3.529	0.06
< 27%	25	95.8	89.8		89.9		
≥ 27%	26	90.2	68.6		11.4		
Lesion Location						0.741	0.389
Spinal Cord	19	88.5	71.5		71.5		
Intracranial	45	94.7	76.3		33.0		
Diagnosis Method						0.869	0.351
Biopsy	23	89.3	74.4		74.4		
Resection	41	94.7	87.9		31.6		
Concurrent Chemotherapy						0.488	0.485
Yes	35	90.8	82.9		44.2		
No	29	96.2	66.3		47.4		

### Cox proportional hazards regression multivariate analysis

The univariate analysis was conducted on statistically significant factors and those nearing statistical significance, as well as factors that may have a clinical impact on prognosis. These were then incorporated into the regression model. The results indicated that age is an independent prognostic factor in patients undergoing radiation therapy for DMG. Younger patients had a poorer prognosis. Please refer to Table 2 for details.

**Table 2:** Results of multivariate analysis

	B	SE	Wald	df	P	Exp(B)	95.0% Exp (B) CI	
							lower	Upper
Gender	0.029	0.542	0.003	1	0.958	1.029	0.356	2.976
Age (Years)	1.660	0.625	7.061	1	0.008	5.258	1.546	17.884
Lesion Location	0.417	0.644	0.419	1	0.517	1.518	0.429	5.365
Diagnosis Method	-0.426	0.674	0.399	1	0.527	0.653	0.174	2.446
Chemotherapy	0.861	0.587	2.151	1	0.142	2.365	0.749	7.475
Histopathological Grading	0.139	0.386	0.130	1	0.719	1.149	0.540	2.446
Ki67 index	0.463	0.397	1.357	1	0.244	1.588	0.729	3.460

## Discussion

Diffuse midline glioma (DMG) is the sole tumor type for which grading is determined through a combination of molecular profiling and histopathological alterations. These tumors are commonly located in midline structures such as the thalamus, brainstem, and spinal cord [6]. Less common sites include the third ventricle, hypothalamus, pineal region, and cerebellar hemispheres [7].The patients in this study were primarily located in the thalamus, brainstem, and spinal cord, consistent with the literature reports. The clinical manifestations of DMG primarily depend on the location of the tumor. The clinical manifestations of the patients in this study included blurred vision, limb weakness, strabismus, headache, and dizziness, which are consistent with the literature reports [8].

In the diagnosis of the patient cohort under study, the criteria included radiological evidence of diffuse infiltrative growth centered on midline structures. The determination of H3K27M mutation status was conducted using immunohistochemistry (IHC), which facilitates the identification of mutations, particularly in the diagnosis of H3K27M-mutant diffuse midline gliomas. Multiple studies have reported a significant correlation between the expression of the H3K27M protein and the presence of H3K27M mutations [9]. The histopathological spectrum of DMG with H3K27M alterations is broad, primarily characterized by astrocytic differentiation. It can manifest in any form ranging from WHO grade 2 diffuse astrocytoma to WHO grade 4 glioblastoma multiforme (GBM), or multiple forms can coexist in different regions. There is no significant difference in the histological grade distribution between adult and pediatric patients [10, 11].Solely relying on histopathological grading to predict patient prognosis has its limitations. In our study group, the histopathological types of tumors included diffuse astrocytoma (WHO grade 2, 9 cases), anaplastic astrocytoma (WHO grade 3, 12 cases), and GBM (WHO grade 4, 24 cases). Some patients did not receive specific histopathological subtyping due to variations in pathological standards across different regions. Upon univariate and multivariate analysis, the histopathological type did not significantly impact prognosis, with no statistical significance observed.

The treatment of gliomas has entered the era of molecular profiling, with pivotal genomic studies highlighting the significance of molecular markers such as *IDH* mutations, 1p/19q codeletion, *MGMT* promoter methylation, *ATRX* mutations, *TERT* promoter mutations, *PTEN* mutations, and *TP53* mutations and so on. These studies have revealed that diffuse intrinsic pontine gliomas (DIPGs) are driven by somatic mutations in the histone H3 gene, defining subgroups with distinct biological and clinical phenotypes and prognoses. Various indicators have differential impacts on the prognosis of gliomas [12]. The Ki67 index serves as an indicator of tumor proliferation rate. In the context of DMG, although the prognostic utility of conventional histological grading alone is limited, the Ki-67 index has demonstrated prognostic value, with similar findings reported in other gliomas [13, 14]. Studies have shown a strong correlation between higher Ki-67 indices and larger tumor volumes in glioblastoma multiforme [15]. Furthermore, elevated Ki-67 indices in DMG are associated with a higher frequency of hypermutated alleles, which in turn correlates with poorer survival rates [16]. In our study, patients were stratified based on a median Ki67 index of 27%, with those exhibiting higher indices tending to have shorter survival periods, although this did not reach statistical significance. These findings suggest that DMGs exhibit considerable heterogeneity, with a multitude of prognostic factors influencing outcomes. Further research is warranted to refine potential markers for study.

Patients with DMG typically exhibit radiological features characterized by diffuse infiltrative growth, accompanied by varying degrees of enhancement, edema, necrosis, and hemorrhage. The imaging characteristics of adult DMG have yielded divergent results across several studies [17–20]. To date, no distinct structural imaging features have been identified, which may be attributed to their lower incidence and the recent recognition of DMG as a separate entity [20]. The highly variable appearances on MRI are largely due to the heterogeneity of tissue pathology, which in turn reflects the histopathological diversity of DMG [19]. The radiological findings in our cohort are consistent with those reported in the literature, with no unique imaging features observed.

Surgical resection is a critical initial treatment modality for gliomas, with higher rates of resection correlating with increased survival [21–23]. Liu et al. [24] collected data from 529 patients with brainstem gliomas and found that the group with complete resection had the highest overall survival rate. In the subgroup of children with low-grade brainstem gliomas (BSG), those who underwent complete resection had a significantly higher survival rate compared to those who did not. However, in adults with low-grade BSG and children with high-grade BSG, the survival rates were higher in the complete resection group, but the differences were not statistically significant. Clinical studies indicate that patients with focal low-grade brainstem and dorsally exophytic tumors may benefit from surgical resection [25]. Ius et al. [26] reported that the incidence of complications following biopsy and surgical resection for high-grade brainstem gliomas was 10.5% and 35.5%, respectively, *p* = 0.009, indicating a statistically significant difference. For patients with DMG, due to the location of the lesion in the midline structures of the brain and spinal cord, the difficulty of surgical resection makes the impact of the extent of tumor removal on prognosis unclear. Karemann and colleagues assessed 85 pediatric patients with DMG and found that the extent of resection was not associated with prognosis [27]. The HERBY trial, which included 42 patients with thalamic DMGs, demonstrated an association between maximal tumor debulking or near-total resection and extended overall survival [28]. The aforementioned studies highlight the impact of anatomical location on the completeness of surgical resection and the probability of postoperative complications, both of which may influence the prognosis of DMG patients. Further refined research is necessary to screen and select patients suitable for surgery to ascertain the role of surgical intervention in the treatment of DMGs. In this cohort, the extent of surgical resection was not fully documented, only biopsy and surgical procedures were stratified for analysis, with no statistical difference observed.

When complete surgical resection is not feasible, conventional high-dose fractionated radiotherapy and temozolomide chemotherapy are the primary treatment options [29, 30]. However, comparisons of different treatment regimens in children and adults, such as concurrent chemoradiotherapy, post-radiotherapy chemotherapy, radiotherapy alone, or chemotherapy alone, have not demonstrated statistically significant advantages [17, 29, 31]. Therefore, treatment approaches should be tailored to each individual case [29]. The overall prognosis for DMG is poor, and improving the survival of patients with DMG is an increasingly researched topic. Radiotherapy remains the mainstay of treatment and its efficacy is well-established. A study by Othman Bin-Alamer et al. [32] found that the survival time of patients who received radiotherapy was significantly longer than those who did not, with a *P* < 0.019. Given the importance of radiotherapy in the treatment of DMG, it is essential to investigate prognostic factors associated with it.

In their comprehensive description of the characteristics of DMG, Carlos et al. [33] reported a generally poor overall survival for the condition, with a median survival duration of 9–12 months in children and 9–19 months in adults. Studies comparing prognostic differences between the two age groups indicate that pediatric patients have nearly identical poor outcomes [34] or even worse prognoses [29] when compared to adults. DMG is common in children and adolescents but can also occur in middle-aged and elderly individuals, with no significant gender differences [34]. Literature reports indicate that the average age at diagnosis ranges from 25 to 39.1 years [35–37]. In a survival analysis conducted by Yao et al. [38] on 33 patients with DMG, it was observed that survival duration increased with age. Notably, patients older than 45 years exhibited a significantly better prognosis than those younger than 19 years (*P* = 0.001). However, Cox regression analysis did not confirm this result as statistically significant. The age range of the patients in this study was 6–56 years, with a noticeable trend across all age groups. After stratifying by the median age of 32 years, both univariate and multivariate analyses were conducted, all of which were statistically significant. This suggests that age is an independent prognostic factor for patients undergoing radiation therapy for DMG.

The therapeutic efficacy of the chemotherapeutic agent temozolomide in the treatment of glioblastoma continues to emerge, as exemplified by a clinical study conducted by the European Organisation for Research and Treatment of Cancer (EORTC). Stupp R and colleagues [39, 40] analyzed data from 573 patients with glioblastoma, finding that the median survival was significantly longer in the group receiving concurrent radiotherapy and chemotherapy (14.6 months) compared to the radiotherapy-only group (12.1 months). This has led to an increasing number of scholars advocating for the use of temozolomide in the treatment of glioblastoma. However, research by Julia R et al. [41] on the concurrent chemoradiotherapy with temozolomide in pediatric patients with diffuse intrinsic pontine glioma did not yield meaningful results. Temozolomide has also been employed as a first-line treatment for diffuse midline glioma (DMG), yet its efficacy has been suboptimal in several studies [29–31]. TMZ not only exerts cytotoxic effects but also possesses a variety of immunomodulatory functions. It can activate compensatory release of cytokines, lower the activation threshold and proliferation of T cells, thereby eliciting a more robust immune response. Despite the potential benefits of chemotherapy, the therapeutic efficacy is limited due to the integrity of the blood-brain barrier (BBB) in most cases of DMG [30, 32]. Compared to radiotherapy alone, TMZ has not demonstrated improvement in tumor burden or overall survival, and is associated with an increased risk of side effects and toxicity. In the absence of other suitable chemotherapy options, TMZ may be considered as a first-line chemotherapeutic regimen for DMG patients during and post-radiotherapy. In our study, patients who received concurrent temozolomide chemotherapy during radiotherapy had mild and tolerable side effects, but no survival advantage was demonstrated. This may be related to the retrospective nature of the study and an imbalance in case selection. Whether it can provide value for patients with higher histopathological grades requires further research with a larger sample size.

Our study has certain limitations. Firstly, due to the accessibility of medical records, patient imaging and surgical data were incomplete. The inclusion of patients from various institutions, which have differing levels of recognition and understanding of DMG, as well as disparities in molecular pathology capabilities, resulted in incomplete acquisition of certain molecular markers, precluding their evaluation. Future work will involve the standardization of assessment criteria for a more in-depth analysis. We plan to standardize the assessment criteria for a more in-depth analysis in the future. Additionally, the small number of cases necessitates the expansion of the sample size for a more thorough investigation.

Preliminary research indicates that patient age significantly impacts the survival rate of those undergoing radiotherapy for DMG. Other factors did not appear to confer a survival benefit for patients with DMG. At present, radiotherapy remains the primary treatment modality for DMG. Future research is needed to explore alternative radiotherapy approaches or the use of sensitizing agents to enhance the efficacy of radiotherapy. Currently, there is a burgeoning body of basic and translational research on DMG, providing insights into pathology, disease mechanisms, and novel treatment strategies that may inform clinical trials. These advances hold promise for improving the poor prognosis of DMG patients through combination therapies. We aim to increase the sample size and refine patient categorization to provide reference indicators for the formulation of clinical treatment plans, offering new hope for patients with DMG.

## Data Availability

The data presented in this study are available on request from the corresponding author in an anonymized form after data privacy check. The data are not publicly available due to data privacy regulations.

## Abbreviations

MDG: Diffuse midline glioma
CNS: Central nervous system
GTV: Gross target volume
CTV: Clinical target volume
GBM: Glioblastoma multiforme
IHC: Immunohistochemistry
DIPGs: Diffuse intrinsic pontine gliomas
BSG: Brainstem gliomas
BBB: Blood-brain barrier
EORTC: European Organisation for Research and Treatment of Cancer
