# Correction to: Prognostic factors analysis of diffuse midline glioma

**DOI:** 10.1007/s11060-024-04634-1

**Published:** 2024-03-15

**Authors:** Jing Jiang, Wen-bin Li, Shao-wen Xiao

**Affiliations:** 1 Department of Neuro-Oncology, Cancer Center, Beijing Tiantan Hospital, Capital Medical University, Beijing, 100071 China; 2 Department of Radiation Oncology, Beijing Tsinghua Changgung Hospital, School of Clinical Medicine, Tsinghua University, Beijing, 102218 China; 3 Department of Radiation Oncology, Peking University Cancer Hospital & Institute, 52 Fucheng Road, Haidian District, Beijing, 100142 China

**Correction to: Journal of Neuro-Oncology**

10.1007/s11060-024-04605-6

The Funding information section was missing from this article and should have read as follows:

## Funding

Li Wenbin received funding from the Beijing Tiantan Hospital Talent Introduction Program (RCYJ-2020-2025-LWB) and the National and Provincial Clinical Key Specialty Capacity Building Project of 2020.

The original article has been corrected.

